# Bile ductal mucosal dysplasia is a possible risk factor for adenocarcinoma in patients with adenomyomatous hyperplasia of the Vaterian system: a single-centre study from China

**DOI:** 10.1186/s12876-023-03096-5

**Published:** 2024-01-02

**Authors:** Weizheng Liu, Jie Li, Zhanyu Yang, Jianan Jiang, Daxu Zhang, Wenping Lu

**Affiliations:** 1 Faculty of Hepato-Pancreato-Biliary Surgery, The First Medical Center of Chinese PLA General Hospital, Beijing, China; 2 Faculty of Pathology Department, The First Medical Center of Chinese PLA General Hospital, Beijing, China

**Keywords:** Adenomyomatous hyperplasia, Ampulla of vater, Common bile duct, Biliary tract carcinoma, Ampullary carcinoma

## Abstract

**Background:**

The relationship between adenomyomatous hyperplasia of the Vaterian system(AV) and cancer is unclear, some reports suggest that AV is often combined with mucosal glandular dysplasia, but it is not clear whether mucosal glandular dysplasia is a risk factor for carcinogenesis of AV. The aim of this study was to retrospective analysis of role of ductal glandular dysplasia as a risk factor in the development of carcinoma in AV.

**Methods:**

A total of 328 cases who underwent surgery with a final pathological diagnosis of adenomyomatous hyperplasia (AH) in the Chinese PLA General Hospital in BeiJing, China, between January 2005 and December 2021 were retrospectively collected. There were Seventeen cases(5%) in which the lesions were located in the common bile duct as well as the ampulla of Vater, and their clinical (age, sex, etc.), imaging (cholelithiasis, etc.) and pathological data (mucosal glandular dysplasia, etc.) were collected. Clinical data and pathological features of AV with or without mucosal glandular dysplasia were analyzed.

**Results:**

There were 17 out of 328 cases of AH occurring in the Vaterian system (5%). Three of seventeen AV cases were associated with carcinoma (18%). Of three cases, two (12%) with the tumor lesions in the mucosal glands adjacent to the AH (biliary tract cancer and ampullary cancer), and one (6%) with carcinoma developed from AH itself in the ampulla of Vater. All carcinomas had adenomyomatous hyperplasia with nearby mucosal glandular dysplasia (MGD). The percentage of BTC or AC was higher in patients with concurrent AH and MGD compared to AH patients without MGD. The results show tendency toward statistical significance (P = 0.082). This difference was more obvious among AH with severe dysplasia compared to adenomyomatous hyperplasia with mild-moderate dysplasia (P = 0.018).

**Conclusion:**

This study is the first to find that AV is associated with biliary tract cancer and ampullary cancer. In AV, the mucosal glandular dysplasia may be a risk factor for the development of malignancy. The underlying mechanism for carcinogenesis of AV could be AH itself or its secretions stimulating mucosal glands hyperplasia, then mucosal glands dysplasia. AV may be a precancerous lesion.

## Background

Adenomyomatous hyperplasia (AH) is a rare tumor-like inflammatory proliferative disease of the entire gastrointestinal tract, predominantly the gallbladder [1–3]. The cases of adenomyomatous hyperplasia of the Vaterian system (common bile duct and the ampulla of Vater) (AV) are extremely rare and are often reported individually [4–12]. AV is of distinct clinical importance from AH in the rest of the gastrointestinal tract, exhibiting biliary obstruction and tumor-like behavior, and is difficult to distinguish from malignancy preoperatively. Therefore, most cases are pathologically confirmed as AV after radical cholangiocarcinoma resection or pancreatoduodenectomy [2, 13, 14].

The relationship between AV and cancer is unclear. No association has been reported between AV and biliary tract cancer (BTC)/ampullary cancer (AC). There are some reports indicating that AV is often associated with mucosal glandular dysplasia [1, 13–17]. However, it is not clear whether mucosal glandular dysplasia is a risk factor for the malignant transformation of AV.

We identified seventeen cases of AV from our clinical file. In this study, we retrospectively collected data and analysed whether mucosal glandular dysplasia is a potential risk factor for AV to develop BTC and AC.

## Methods

### Study population

The cases with a final pathological diagnosis of AV from January 2005 to December 2021 at the Chinese PLA General Hospital in BeiJing, China, were collected. Clinical data collected in this study included age, sex, smoke (> 30 pack–year) [18] and alcohol abuse (> 80 mg/d) [19], family history of tumors, BMI, cholangitis (symptoms include abdominal pain, jaundice, fever), preoperative laboratory tests suggestive of biliary obstruction (i.e. total bilirubin, direct bilirubin, alkaline phosphatase, and γ-glutamyl transpeptidase), tumor marker CA19-9, surgical approach, and pathological findings from surgical specimens. The patient’s enhanced computed tomography (CT), magnetic resonance cholangiopancreatography (MRCP), and enhanced magnetic resonance imaging (MRI) images were collected to confirm the concurrent bile duct stones and the presence of bile duct dilatation (defined as a maximum diameter of the common bile duct ≥ 10 mm).

The pathological findings were re-confirmed, including the AH, the location of the lesion, the mucosal glandular dysplasia (MGD), and the malignancy tumor, respectively.

### Definition of adenomyomatous hyperplasia (AH)

According to the World Health Organization (WHO) classification, AH is a duct-like structure within hyperplastic smooth muscle [20], characterized by hyperplasia of intramural biliary glands (some cystically dilated) and smooth muscle hyperplasia in the terminal portion of the common bile duct or the wall of the common channel. Typically, proliferating glands without cellular atypia surrounded by irregularly proliferating smooth muscle bundles can be observed [14].

### Definition of mucosal glandular dysplasia(MGD)

Mucosal glandular present with histological atypia or cytological atypia, but not sufficient to diagnose adenocarcinoma, including signs of nuclear schizophrenia, necrosis, and peripheral tissue/nerve/vascular infiltration. (1) cytological atypia: cuboidal or columnar cells with various degrees of nuclear enlargement and pleomorphism. (2) histological atypia: Predominantly flat, micropapillary, pseudopapillary or cribriform architecture [21, 22].

MGD near AH is defined as Glandular near the surface of adenomyomatous hyperplasia appear histological atypia or cytological atypia rather than AH itself.

### Definition of severity of MGD

Mucosal glandular dysplasia can be classified as mild to moderate and severe dysplasia. (1) mild to moderate dysplasia: Lesion involves relatively small areas and does not involve peribiliary glands, hyperchromatic nucleus with relatively regular nuclear membrane, increased nuclear to cytoplasmic ratio, nuclear stratification,preserved nuclear polarity. (2) severe dysplasia: Lesion involves extensive areas, including peribiliary glands, hyperchromatic nucleus with irregular nuclear membrane, very high nuclear to cytoplasmic ratio, relatively extensive degree of polymorphism and prominent nuclear atypia, complex nuclear stratification, loss of nuclear polarit, but not enough to diagnose adenocarcinoma [22].

### Statistical analysis

Measurement data fitting normal distribution were expressed as mean ± standard deviation while data fitting non-normal distribution were expressed as median (Q1,Q3). T-test was adopted for normal distribution and Mann-Whitney U test was used for non-normal distribution when comparing measurement data between the two groups, whereas qualitative data were subjected to Fisher’s exact test for statistical analysis and assessment of differences. P < 0.05 indicated a significant result. All statistical analyses were performed using SPSS 26.0.

## Results

From January 2005 to December 2021, 17 of 328 cases of adenomyomatous hyperplasia at Chinese PLA General Hospital in Beijing, China, occurred in the Vaterian system (5%). The specific clinical features are summarized in Table 1. Twelve patients were men and five women. Their mean age was 58.0 ± 11.3 years. The majority of patients were admitted for examination with significant symptoms. Eight (47%) patients had symptoms of cholangitis, with the main clinical manifestation being abdominal pain in eight (47%), followed by jaundice in seven (41%) and fever in two (12%). Only two (12%) patients had no clinical manifestations before surgery. Imaging studies (MRCP or CT) were positive for bile duct stones in 6 patients (35%). More than half of the patients present with varying degrees of abnormal hepatic function tests and eight (47%) had elevated levels of total bilirubin (> 17.1umol/L).

**Table 1:** Demographic and clinical variables

	*N = 17*
Age(years)	58.0 ± 11.3
Sex ratio (M: F)	12 : 5
Smoke (n [%])	4(23)
Alcohol abuse (n [%])	5(29)
Family history of tumors (n [%])	3(17)
BMI (kg/m^2^)	23.8 ± 2.3
Cholangitis manifestations (n [%]) Abdominal pain Jaundice Fever	8(47) 7(41) 2(12)
Choledocholithiasis (n [%])	6(35)
Laboratory tests Total bilirubin (µmol/L) Direct Bilirubin (µmol/L) Alkaline phosphatase (U/L) γ-glutamyl transpeptidase(U/L) CA19-9 (µ/ml)	16.7(8.9,113.5) 3.8(2.7,100.9) 134.4(69.5,300.3) 103.4(28.1,294.1) 12.1(7.6,83.8)
Dilation of the bile ducts (n [%])	15(88)
Surgical approach (n [%]) Radical pancreaticoduodenectomy Radical resection for hilar cholangiocarcinoma	16(94) 1(6)
AH involvement sites (n [%]) Common bile duct Proximal Distal Ampulla of Vater Both CBD and AoV	8(47) 1(6) 7(41) 8(47) 1(6)
Cancer (n [%])	3(18)
Mucosal glandular dysplasia (n [%])	8(47)

AH = adenomyomatous hyperplasia. BMI = Body Mass Index. CBD = Common bile duct. AoV = Ampulla of Vater

Laboratory tests are preoperative

**Fig. 1:**
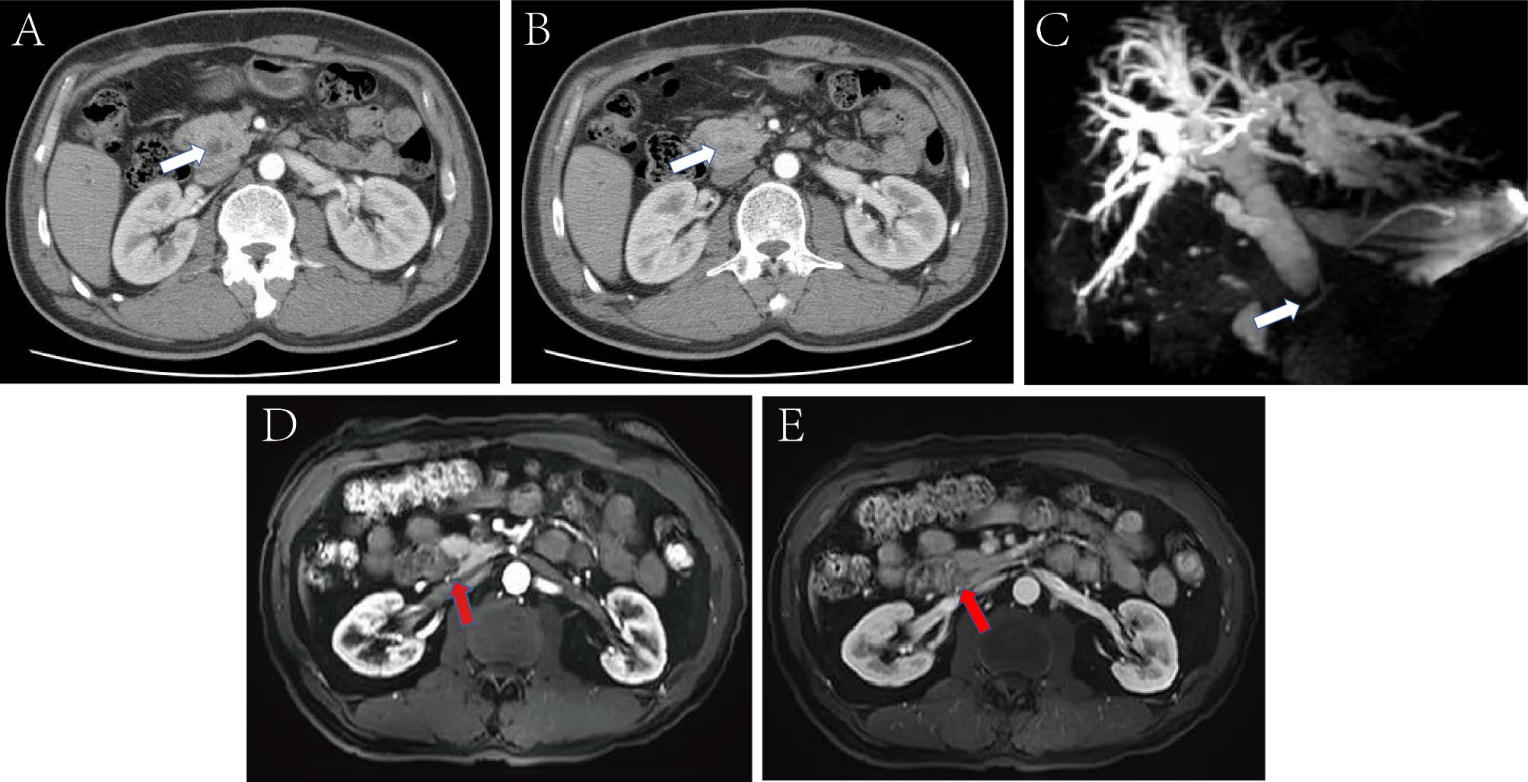
(**A, B, C**) are CT and MRCP images of a 52-year-old patient with AH of the distal bile duct with cholangiocarcinoma: showing marked dilatation of the bile duct and truncation of the distal bile duct (white arrow), with no obvious occupying lesions. (**D, E**) are MRI images of a 66-year-old patient with AH of the distal bile duct: An approximately 16*17 mm abnormal oval signal shadow(red arrow) is seen in the ampulla of Vater, part of the lesion is poorly demarcated from the head of the pancreas, and dynamic enhancement scans show mild enhancement. (**C**) Arterial phase; (**D**) Balanced phase

In ten patients (59%), preoperative imaging showed a lesion at the corresponding site, and in seven patients (41%) there was an abnormally dilated extrahepatic and intrahepatic bile duct without obvious lesions (Fig. 1). Detailed imaging findings of AV are shown in Table 2. All patients in this study did not perform endoscopic retrograde cholangiopancreatography, endoscopic ultrasonography and intraductal ultrasonography including biopsy before surgery. Five patients (29%) showed elevated levels of serum CA 19 − 9 (> 37u/ml). All patients were highly suspected of malignant tumor and received surgical treatment. Sixteen patients (94%) underwent radical pancreaticoduodenectomy and one patient (6%) received radical resection for hilar cholangiocarcinoma. The postoperative pathology confirmed an AH in one (6%) case in the proximal part of the common bile duct, seven (41%) cases in the distal part of the common bile duct, eight (47%) cases in the ampulla of Vater, and one (6%) case simultaneously in both sites.

**Table 2:** Imaging features of adenomyomatous hyperplasia of the Vaterian system

	Age/Sex	Examination methods	Imaging features
Case 1	52/M	CT/MRCP	No lesion/No lesion
Case 2	50/M	CT/MRCP	15 mm-sized oval enhanced mass was detected in portal bile duct/No lesion
Case 3	73/M	CT	No lesion
Case 4	66/M	MRI	16 mm-sized oval enhanced mass was detected in AoV
Case 5	57/F	CT/MRCP	13 mm-sized oval enhanced mass was detected in AoV/12 mm low signal mass was detected in AoV
Case 6	54/F	CT	No lesion
Case 7	67/M	CT	14 mm-sized oval enhanced mass was detected in AoV
Case 8	69/M	CT	16 mm-sized oval enhanced mass was detected in AoV
Case 9	49/M	MRI	No lesion
Case 10	66/M	MRI	18 mm-sized oval enhanced mass was detected in AoV
Case 11	65/M	MRI	18 mm-sized oval enhanced mass was detected in distal bile duct
Case 12	54/M	CT/MRCP	No lesion/No lesion
Case 13	63/F	CT	10 mm-sized oval enhanced mass was detected in distal bile duct
Case 14	38/M	MRI/MRCP	No lesion/No lesion
Case 15	34/M	MRI/CT/MRCP	14 mm-sized oval enhanced mass was detected in AoV/No lesion/No lesion
Case 16	64/F	MRI	Circumferential enhancement was detected in distal bile duct
Case 17	72/F	CT	No lesion

M = male. F = female. AoV = Ampulla of Vater

**Fig. 2:**
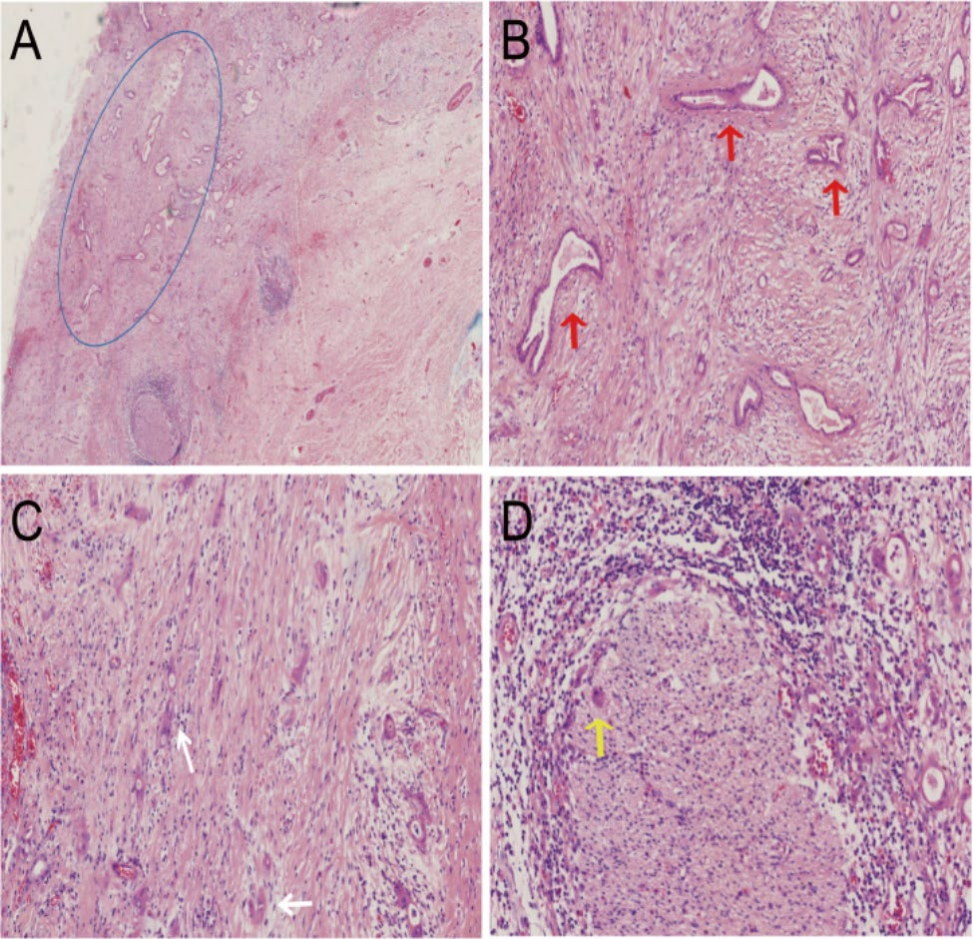
AH in ampulla of Vater transformation into a moderately differentiated adenocarcinoma (blue oval). Infiltrative growth of cancerous tissue in the duct wall (**A**); irregular tumor glandular structure, variable size glandular lumen (red arrow), infiltrative growth of individual cells, and mucus visible within some individual cells with signet-ring cell-like features (white arrow) (**B**, **C**), and tumor cell infiltrating nerve fibers (yellow arrows) (**D**)

Carcinoma was found in three of seventeen AV cases (18%), two were in the mucosal glands of the duct wall adjacent to the AH, and one was adenocarcinoma arising from AH (Fig. 2).

**Fig. 3:**
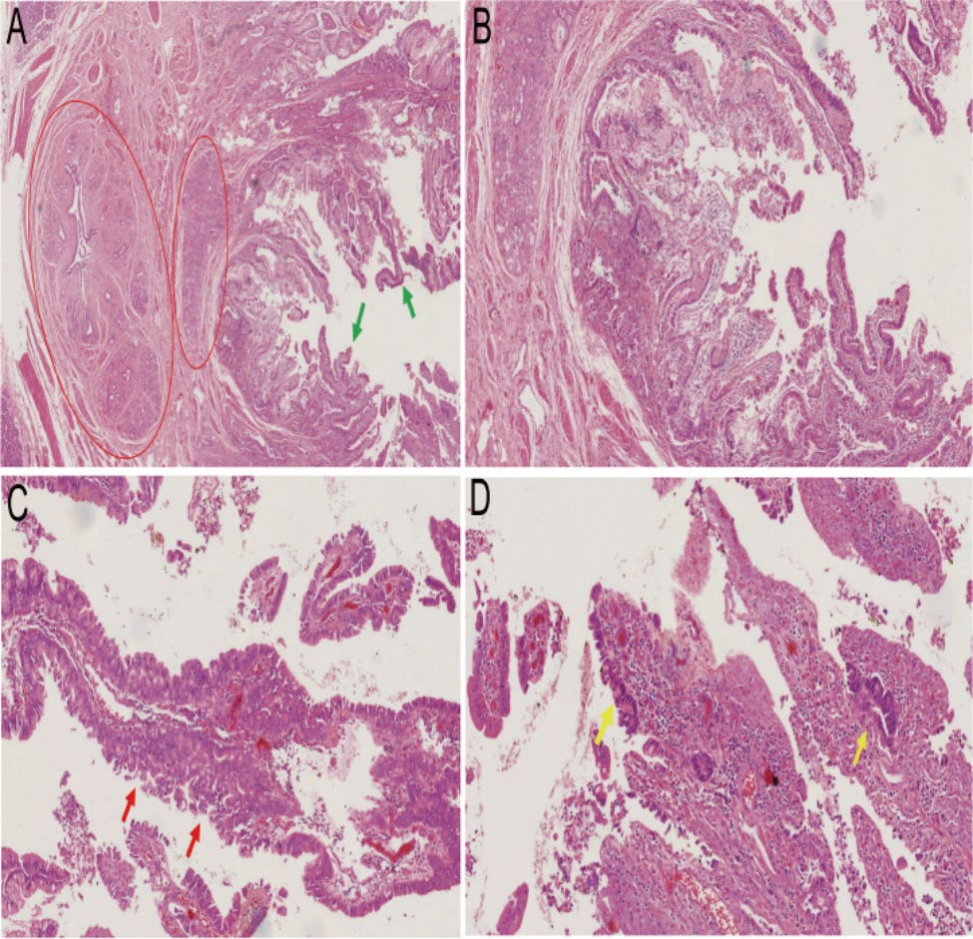
Papillary hyperplasia of dysplasia glands (green arrow) on the surface of AH in common bile duct(red oval), with a clear boundary between AH and surface dysplasia glands (**A**); papillary structure of surface dysplasia glands with small vascular hyperplasia and lymphocytic infiltration visible in the papillary axis (**B**); surface dysplasia glands with complex papillary structures, visible multilevel papillary branches, multiple cell layers, and glandular fusion features, exhibiting sieve-like papillary structures(red arrow) with cuboidal cells and moderate cellular dysplasia (**C**); degenerative tissue necrosis visible on the surface of the papillae in some areas, with moderate cellular dysplasia glands and nested clusters of cells with severe dysplasia visible around the necrotic material (yellow arrow) (**D**)

In all cases of carcinoma(n = 3,18%), mucosal glands dysplasia near the AH were observed (Fig. 3). In contrast, patients without mucosal glands dysplasia in the duct wall did not develop carcinoma. The percentage of BTC or AC was higher in patients with concurrent AH and MGD (AH-MGD) compared to AH patients without MGD (AH-non-MGD), the result was tendency toward statistical significance(37% vs. 0%, P = 0.082; Table 3). This difference was more marked among AH with peripheral mucosal glands severe dysplasia of the duct wall (AH-severe-MGD) compared to AH with adjacent mucosal glands mild-moderate dysplasia of the duct wall (AH-mild-moderate-MGD) (100% vs. 0%, P = 0.018).

**Table 3:** Clinical features of AH-MGD and AH-non-MGD groups

	AH-MGD n = 8(47%)	AH-non-MGD n = 9(53%)	*P value*
Sex ratio (M:F)	6:2	6:3	1.000
Age (years)	61.0 ± 8.7	56.0 ± 13.3	0.391
Smoke (n [%])	2(25)	2(22)	1.000
Alcohol abuse (n [%])	3(37)	2(22)	0.620
Family history of tumors (n [%])	0	3(33)	0.206
BMI (kg/m^2^)	24.7 ± 1.5	23.0 ± 2.7	0.137
Choledocholithiasis (n [%])	3(37)	3(33)	1.000
Dilation of the bile ducts (n [%])	8(100)	7(78)	0.471
Cholangitis (n [%])	4(50)	4(44)	1.000
Cancer (n [%])	3(37)	0	0.082^*^
Laboratory tests Total bilirubin (µmol/L)	18.6(13.5,119.0)	9.3(7.4,132.1)	0.336
Direct Bilirubin (µmol/L)	4.8(3.6,107.3)	3.6(1.8,114.6)	0.470
Alkaline phosphatase (U/L)	150.4(82.4,336.4)	83.3(64.6,277.9)	0.290
γ-glutamyl transpeptidase (U/L)	127.0(29.8,297.4)	98.30(23.0,400.5)	0.630
CA19-9 (µ/ml)	13.4(8.8,103.1)	10.0(6.3,73.4)	0.336

AH-MGD, adenomyomatous hyperplasia with nearby mucosal glandular dysplasia

AH-non-MGD, adenomyomatous hyperplasia without nearby mucosal glandular dysplasia. BMI, Body

There were no statistically significant differences (p > 0.05) in age, sex, occurrence of bile duct stones, cholangitis, biliary obstruction and laboratory indicators related to obstruction in (AH-MGD) (n = 8,47%) compared to (AH-non-MGD) (n = 9,53%) (Table 3).

## Discussion

A total of seventeen cases of AV were collected in this study, which is the largest series to date. About to half of the cases were adjacent to the AH in MGD (47%). In contrast, the presence of AH-MGD in this study was not associated with irritation from cholangitis or obstruction due to stones or lesions (p > 0.05). The findings of the study suggest that AH-MGD (particularly AH-serve-MGD, p = 0.018) is associated with BTC or AC, although p value was very close to 0.05 (37% vs. 0%, p = 0.082). We speculate that AH contributes by some mechanism to mucosal glands dysplasia of the duct wall, or even to severe dysplasia or carcinoma.

The preoperative diagnosis of AV is challenging [15]. It usually presents signs of biliary obstruction and cholestasis and often shows non-specific clinical manifestations such as abdominal pain and jaundice, or may be asymptomatic [14]. Preoperative imaging (CT, MRI, MRCP) often shows biliary obstruction or tumor-like masses [14, 15]. Approximately 9% of patients are diagnosed preoperatively or intraoperatively with AV [2]. Most patients underwent surgical resection when malignancy could not be excluded, and the diagnosis was eventually confirmed by pathology. All seventeen cases of AV in this study were misdiagnosed as BTC or AC and were unexpectedly identified as AV on postoperative pathology.

In this study, AH-MGD was observed in eight cases (47%), Notably, we have found AH combined with mucosal or mucosal glandular dysplasia in other reports [1, 13–17]. Although, cholelithiasis or inflammation are thought to cause dysplasia in the biliary system [23–26], the findings of this study showed no statistically significant difference between AH-MGD and AH-non-MGD in the occurrence of bile duct stones, cholangitis, or biliary obstruction, and no association of AH-MGD with bile duct stones, cholangitis irritation or obstruction. We hypothesize that (1) the mucosal glands of the duct wall are stimulated by AH develop papillary hyperplasia, then dysplasia. (2) The mucosal glands of the duct wall are impacted by the secretion produced by AH, or perhaps the presence of AH alters the function of the smooth muscle of the duct wall, resulting in dysplasia. In the present study of AV combined with malignancy, two cases (12%) had malignant lesions located in the mucosal glands of the duct wall near the AH, and serve dysplasia of mucosal glands were seen around the AH, such characteristics should confirm the conjecture of the present study.

Although AV is rare and has mostly been reported as single cases [4–12]. we identified three cases with malignant transformation among the seventeen cases of AV. Nevertheless, in gallbladder AH, the incidence of gallbladder carcinoma is 1–12% [27–29], hence the AH itself carries some risk of carcinogenesis. All cases of carcinoma presented with AH-MGD, whereas those AH-non-MGD were not found to have comorbid malignancy. This distinction was particularly pronounced in patients with AH-serve-MGD (100% vs. 0%, P = 0.018). AH incorporating peripheral mucosal glands dysplasia, especially severe dysplasia, may be associated with BTC or AC, although the p-value did not meet statistical criteria (p = 0.082), which is thought to be a result of the small sample size. Our results suggest that dysplasia of mucosal glands in the duct wall, especially severe dysplasia, may be correlated with the process of AH leading to malignancy in the Vaterian system, which may be a precancerous lesion.

## Conclusions

In summary, for the first time, we have identified AV combined with carcinoma. The risk factor may be the mucosal glandular dysplasia. It is hypothesized that the potential mechanism of carcinoma in AV is AH itself or its secretions causing glandular hyperplasia, dysplasia, and then carcinoma. AV may be a precancerous lesion. Due to the sample, the relationship between AH, dysplasia, and carcinoma needs to be further studied. In the future, we will need a larger sample size from multi-center studies in multiple medical centers to elucidate this relationship and Additional molecular biology studies are needed to clarify the mechanism of adenomyomatous hyperplasia leading to mucosal glandular dysplasia and malignancy.

## Data Availability

The datasets used and analysed during the current study are available from the corresponding author on reasonable request.

## Abbreviations

AH: adenomyomatous hyperplasia
AV: adenomyomatous hyperplasia of the Vaterian system
MGD: mucosal glandular dysplasia
BTC: biliary tract cancer
AC: ampullary cancer
Aov: Ampulla of Vater
